# The pig as a preclinical model for human phage therapy of polybacterial enteric infections

**DOI:** 10.3389/fmicb.2026.1857948

**Published:** 2026-08-07

**Authors:** Joan Colom, Abiyad Baig, Lajos Kalmar, Aouatif Belkhiri, Annabel Travers, Adriano M. Gigante, Phillippa L. Connerton, Ian F. Connerton, Andrew J. Grant, Mark Holmes, Paul A. Barrow, Robert J. Atterbury

**Affiliations:** 1 School of Veterinary Medicine and Science, University of Nottingham, Sutton Bonington, Leicestershire, United Kingdom; 2 MRC Toxicology Unit, Cambridge, United Kingdom; 3 School of Biosciences, University of Nottingham, Sutton Bonington, Leicestershire, United Kingdom; 4 Department of Veterinary Medicine, University of Cambridge, Cambridge, United Kingdom

**Keywords:** bacteriophage therapy, *Campylobacter*, *Escherichia coli*, Salmonella, antimicrobial resistance, polymicrobial infection, swine, gut microbiota

## Abstract

**Background:**

Diarrhoeal disease remains a leading cause of morbidity and mortality among children in low-and-medium-income countries (LMIC). Polybacterial infections and increasing antimicrobial resistance complicate treatment, underscoring the need for alternative approaches. Bacteriophage therapy offers a targeted treatment capable of combating multiple pathogens simultaneously. Here, we report use of a bacteriophage cocktail targeting Enterotoxigenic *E. coli*, *Salmonella* and *Campylobacter* in a polybacterial porcine model of infant enteric disease.

**Methods:**

Weaned pigs were challenged with all three pathogens and subsequently treated with a cocktail of three phages. Pathogen loads, phage titres, clinical scores and fecal microbiome composition were measured and compared with untreated pathogen-challenged controls and unchallenged sentinel animals.

**Results:**

Phage-treated pigs exhibited significant reductions in *E. coli* and *Salmonella* burdens, each decreasing by >1.8 log_10_ CFU/g relative to unchallenged controls (*p* < 0.05). Treatment produced corresponding increased in bacteriophage titres and led to significant improvements in clinical scores. Conversely, *Campylobacter* loads remained largely unchanged following phage administration. Microbiome analysis showed differences between challenged and unchallenged pigs (*p* = 0.09), and between pathogen-challenged and phage-treated pigs (*p* = 0.5), although these were not statistically significant.

**Conclusion:**

This study demonstrates the first use of phage therapy to target polybacterial enteric infections in a pig model of human disease. Simultaneous reductions in pathogen load and improved clinical outcomes highlight the potential of bacteriophage cocktails as treatments for bacterial enteric infections of relevance to children in low and middle-income countries, and to livestock health.

## 1 Introduction

Diarrhea remains a major cause of death worldwide although there has been a significant reduction in the number of cases over the last few decades (GBD 2016 Diarrhoeal Disease Collaborators, 2018; UNICEF, 2022). In the early years of this century in low to medium income countries (LMIC), moderate to severe diarrhea (MSD) caused ~444,000 (9%) of deaths in children 5 years old or less (UNICEF, 2022). MSD is typically multifactorial, involving pathogens such as *Escherichia coli*, *Campylobacter*, *Salmonella* and *Shigella* (Kotloff, 2022; Platts-Mills et al., 2018). Rotavirus and norovirus are also frequently involved (Black et al., 2024). There is some indication that predominance of bacterial species may vary between continents with *Campylobacter* and *Shigella* in Asia and *E. coli* in Africa and America (Sayeed et al., 2025). MSD is inexorably linked with socio-economic status, the living environment, education level and accessibility of health care services (Ashaolu et al., 2025; Yu et al., 2025). Co-morbidities with respiratory infections are not uncommon in LMIC with similar risk factors (Tekeba et al., 2025).

Few effective vaccines are available covering the major pathogens. Treatment usually involves oral rehydration therapy, which prevents dehydration but does not reduce pathogen shedding. Antimicrobial therapy is not recommended by the WHO for non-invasive/non-bloody diarrhea due to concerns about increasing antimicrobial resistance (AMR), Aboderin et al., (2010). Indeed, it can be futile if the strains involved are already AMR and may thus contribute to increased childhood morbidity and mortality (Bernabé et al., 2017; Djie-Maletz et al., 2008; Haghi et al., 2014; Konaté et al., 2017) and associated increased health care costs.

AMR is now regarded as a major existential threat to human and animal health.^1^ Human deaths directly attributable to AMR are already estimated to be in excess of 1 million p.a. (Antimicrobial Resistance Collaborators, 2022) with one older report (Review on Antimicrobial Resistance, 2016) arguing that AMR-associated mortality could reach 10 million p.a. by 2050, affecting global GDP by 2–3.5%, and these figures are likely to be a gross underestimate. AMR is widespread among enteric bacterial pathogens in LMIC, particularly multidrug-resistant strains of *E. coli*, *Shigella* and *Salmonella* (Neupane et al., 2023; Shakoor et al., 2019; Tait, 2020).

Human society has reached a critical juncture regarding AMR, recognized for many years by international institutions including the WHO (World Health Organization, 2015; European Commission, 2017; Willemsen et al., 2022) and, in the context of One Health, including Food and Agriculture Organization (FAO), World Organization for Animal Health (WOAH) (founded as OIE) and United Nations Environment Programme (UNEP). They recognize the problems of overuse of antibiotics in LMIC, the importance of improved and rapid diagnosis, improved vaccination and reduction in use of antibiotics including the complete withdrawal of their use for growth stimulation. However, these measures will not lead to a rapid reduction in AMR in bacterial strains which are already resistant, and international institutions have called for research into novel approaches to controlling AMR infections, including the use of bacteriophage (OECD, 2022; Pelfrene et al., 2016; Review on Antimicrobial Resistance, 2014).

Lytic bacteriophages (phages), show enormous promise as alternative treatments, demonstrating safety and efficacy in several animal species (Gómez-Ochoa et al., 2022). These include cholera in rabbits (Bhandare et al., 2019) and enteritis in livestock (Smith and Huggins, 1983) both of which studies showed reduced fecal shedding of the pathogen in addition to leading to clinical improvement. A clinical trial treating canine otitis externa has also been successful (Hawkins et al., 2010). The international STAR-IDAZ consortium has recently reviewed the application of bacteriophages in a One Health context and have developed a outcome-derived roadmap toward practical application (Atterbury et al., 2026). This also includes the employment of bacteriophages to specifically target bacterial antibiotic resistance rather than the bacterial cell (Colom et al., 2019; Ojala et al., 2013). One identified key hurdle was the use of appropriate animal models of human and other bacterial diseases. Domestic pigs are an effective model for phage therapy because of the similarity to humans of their physiology (Meurens et al., 2012) and pathogen susceptibility (Holland, 1990).

Here, we assess the efficacy of a phage cocktail targeting three enteric pathogens (*E. coli*, *Salmonella* and *Campylobacter*) in a polybacterial infection of pigs. We evaluate the impacts on pathogen shedding, clinical outcomes and the fecal microbiome. To our knowledge, this is the first study to use phage therapy against polybacterial infections in a porcine model of human enteric disease. We hypothesized that phage therapy would reduce pathogen colonization and improve clinical outcomes without causing significant disruption to the gut microbiome.

## 2 Materials and methods

### 2.1 Bacterial strains and phage

Enterotoxigenic *E. coli* (ETEC) P433 Rif^R^ (Smith and Huggins, 1983) and *Salmonella* Typhimurium 4/74 Nal^R^ (Morgan et al., 2004) were cultured in Luria Bertani (LB) broth or agar (LA) (1% w/v, Sigma). *C. coli* LL-SB7 (isolated from a sow in 2013) was cultured in Nutrient Broth No.2 (NB2, Oxoid, Basingstoke, United Kingdom) and Mueller Hinton Agar (Thermo Fisher). Bacterial enumeration used Phosphate-Buffered Saline (PBS) as a dilution buffer. Selective bacterial enumeration used Brilliant Green Agar (BGA, Thermo Fisher) with 100 μg/mL sodium nalidixate (Sigma) for *S.* Typhimurium, MacConkey agar (Sigma) with 100 μg/mL rifampicin for ETEC, and modified Charcoal Cefoperazone Deoxycholate agar (mCCDA) with supplement SR0155 for *C. coli* (Oxoid). Plates for *Campylobacter* culture were incubated for 48 h at 41.5 °C under microaerobic conditions (5% O_2_, 5% H_2_, 10% CO_2_, 80% N_2_). Plates for ETEC and *Salmonella* were incubated aerobically at 37 °C for 24h.

The isolation of phage vB_EcoP_P433 (P433.1) and vB_SenM_10 (Phi10) have been described previously (Atterbury et al., 2007; Smith and Huggins, 1983). Phage LL-A6 was isolated from pigs using host *C. coli* NCTC12667 (Bryner et al., 1973). Phage lysates were prepared by infecting mid-exponential cultures (~8 log_10_ CFU/mL) at MOI 0.1 and incubating at 37 °C (ETEC and *Salmonella*) or 41.5 °C under microaerobic conditions (*Campylobacter*), shaking at 100 rpm, overnight or until lysis. Lysates were centrifuged at 5,000 × *g* for 10 min and filtered (0.22 μm pore-size, Millipore). Phage titres were determined by serial dilution in SM buffer (50 mM Tris-Cl pH7.5, 0.1 M NaCl, 8 mM MgSO_4_·7H_2_O and 0.01% gelatin) followed by standard double-agar overlay assays using LB or NB2 agar (0.5% w/v) seeded with 0.1 mL (0.3 mL for *Campylobacter*) of an overnight culture. Lysates were stored at 4 °C until required. These were then filtered using a 0.22 μm filter and re-titrated prior to administering each dose.

### 2.2 Host range profiles

The bacteria (*n* = 29, Supplementary Table S1) were cultured on Tryptone Soy Agar (TSA) for 24 h at 37 °C or Mueller Hinton agar for *Campylobacter.* Cell suspensions (500 μL of ~8 log_10_ CFU/mL in 10 mM MgSO_4_) were mixed with 5 mL molten TSA top agar and poured onto TSA /Mueller Hinton agar plates to produce lawns. Once the lawns had dried, phage suspensions (7.7-8 log_10_ PFU/mL) were serially diluted and spotted (10 μL) in triplicate onto the surface. Plates were incubated at 37 °C (41.5 °C for *Campylobacter*) for 24 h before examining for plaques (Baig et al., 2017).

### 2.3 Susceptibility of phage in *in vitro* simulated gastric conditions

Phage susceptibility to simulated gastric fluid (SGF) was determined as described previously (Dlamini et al., 2023). Briefly, 1 mL of each phage was separately added to 8.9 mL volumes of SGF (pH 2.0, 3.2 mg/mL pepsin, Sigma-Aldrich/Merck, 0.5% NaCl) containing 0.1 mL distilled water (no-CaCO_3_ control) or 0.1 mL sterile CaCO_3_ suspension (20% w/v). Samples were incubated (37 °C, 150 rpm, 1 h); negative controls used SM buffer instead of SGF. Post-incubation titres were determined by spotting serial dilutions of the phage suspensions onto double agar overlays as described above.

### 2.4 One-step growth curve of bacteriophages

Mid-exponential cultures of ETEC P433, *S.* Typhimurium 4/47 and *C. coli* LL-SB7 (9.9 mL) were infected separately with phage P433.1, Phi10 or LL-A6 at respectively at a MOI of 0.01. Following 5 min adsorption, samples (0.1 mL) were diluted into 9.9 mL of MH broth. Samples were collected at regular intervals and plated for PFU enumeration by double-agar overlay after 24 h incubation at 37 °C (42 °C and microaerobic for *C. coli*).

### 2.5 Phage genome analysis

Raw FastQ files were quality-filtered using Cutadapt and Sickle (Joshi and Fass, 2011; Martin, 2011) before *de novo* assembly (SPAdes, v3.6.2, Bankevich et al., 2012), annotation (Pharokka; Seemann, 2014) and submission to NCBI [BioProject: PRJNA1141915 (LL-A6)], accessions MN384978 and MN384979 (P433.1 and Phi10 respectively).

### 2.6 Enteric disease infection model

Post-weaned (21-day-old) male Large White/Landrace cross pigs were obtained from a commercial farm for this study, which was performed at Drayton Animal Health (Drayton, Stratford-upon-Avon, United Kingdom); a GLP-compliant contract research organization. Experimental design followed ARRIVE guidelines^4^ and performed under UK Home Office Project License P46999B2E, with approval from the University of Nottingham Animal Welfare and Ethical Review Body. Fecal screening on selective agar or overlays, respectively, was used to confirm pigs were free of *Salmonella*, *Campylobacter* or bacteriophages active against the challenge strains prior to starting the experiment.

For *Salmonella* enumeration, fecal samples were homogenized 1:10 (wt:vol) in PBS to which was added an equal volume of double strength Selenite broth (Oxoid) with overnight incubation followed by plating on selective media as described above. Samples for *Campylobacter* isolation were decimally diluted in Maximum Recovery diluent (Oxoid CM 733, Basingstoke, United Kingdom) and plated on Cefaperazone Charcoal Deoxycholate Agar selective medium (Oxoid CM 739) and incubated as described above.

To test for the presence of phages specific for the target bacteria, a suspension of freshly voided feces (1:10, wt/vol) was prepared in SM buffer and subjected to centrifugation at 13,000 × *g* for 5 min to remove bulk debris. The supernatant was then filtered through a 0.22 µm-pore-size filter to remove any remaining bacteria. Filtrate (1 mL) was then added to separate 9 mL volumes of NB2 (for *Campylobacter*) or LB (for ETEC and *Salmonella*) containing 1 mL of a mid-exponential culture of host bacteria. These cultures were incubated for 48 h at 41.5 °C under microaerobic conditions (*Campylobacter*) or aerobically for 24 h at 37 °C for ETEC and *Salmonella,* shaking at 50 rpm. Following incubation, the cultures were filtered (0.22 µm pore size) again before enumerating phage on double agar overlays as described above.

**Figure 1:**
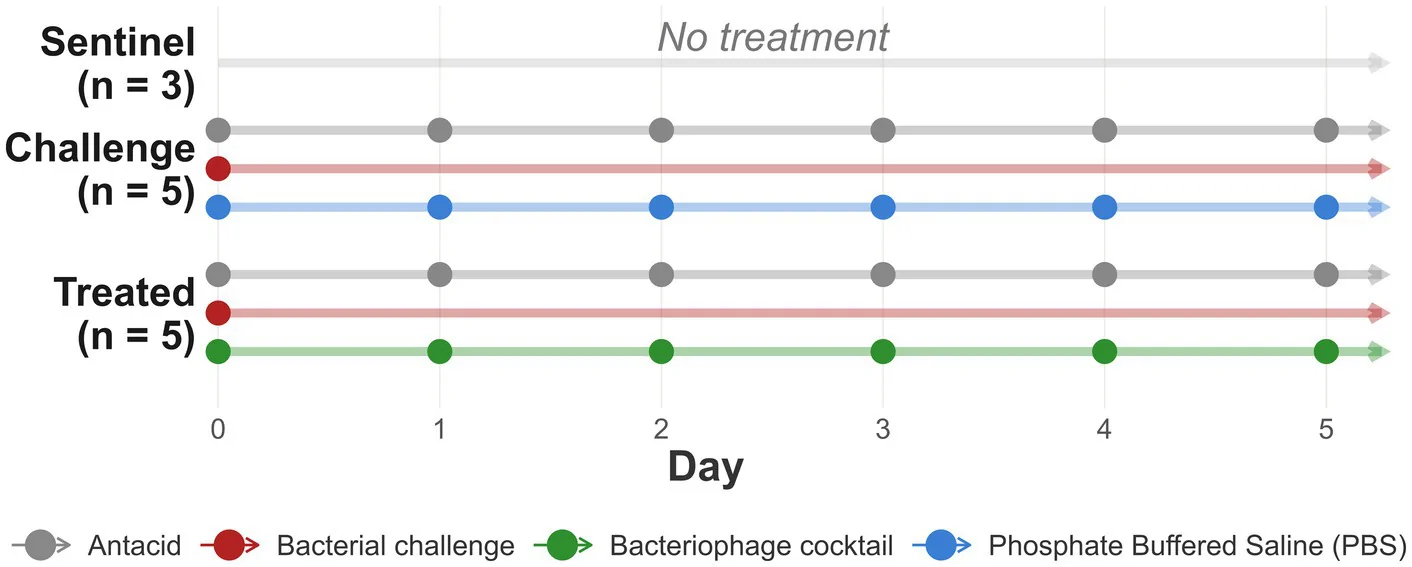
Enteric disease infection model schedule and groups. Pigs were assigned at 23 days of age to Sentinel (*n*=3), Challenge (*n*=5), and Treated (*n*=5) groups. Sentinel pigs remained unchallenged and untreated throughout the trial. Challenge pigs received oral antacid followed by ~9 log_10_ CFU each of ETEC, *S*. Typhimurium, and *C. coli*. Treated pigs received the same antacid and bacterial challenge, followed 4h later by ~10 log_10_ PFU of phages P433.1, Phi10, and LL-A6. Phage treatment was repeated every 24h with antacid until the end of the trial. Challenge pigs received antacid and PBS at the same 24h intervals.

Pigs were then weighed and allocated randomly to one of three groups (Figure 1). Each group was housed in a separate room with appropriate biosecurity changing clothing, boots and gloves between rooms and disinfectant foot baths. All groups were monitored daily for the presence of pathogens and phage.

Feed was withdrawn 16 h before bacterial challenge and 20 h before phage treatment. On day 0 (23 days-old), pigs assigned to the phage-treated group received 3 mL of a 20% w/v CaCO_3_ antacid orally, followed by 10 mL of a suspension containing 9 log_10_ CFU/mL each of *S.* Typhimurium 4/74, ETEC P433 and *C. coli* LL-A6. Four hours later they received another 3 mL of antacid, followed by 10 mL of a phage cocktail comprising P433.1, Phi10 and LL-A6 (each at 10 log_10_ PFU/mL in PBS). Challenge-only pigs received 3 mL of antacid followed by 10 mL of PBS orally. Phage administration, preceded by antacid, was repeated every 24 h until Day 5 with animals in the Challenge-only group receiving antacid and PBS buffer in parallel. Sentinel animals remained untreated.

Clinical observations were conducted daily using a defined scoring system (Supplementary Table S2). Daily weight and rectal temperatures were collected from pigs in all groups. Fecal samples were collected from individual animals daily and homogenized in 5 mL of PBS with sterile fine sand before enumeration of phage and bacteria by spotting decimal dilutions of filtrate or spread plating 0.1 mL volumes onto selective media in triplicate as described above. Shotgun metagenomic sequencing (Illumina, 40 M reads per sample) was performed on fecal samples from individual animals on days 2, 4 and 5. All pigs were humanely euthanized on Day 5 by penetrative captive bolt stunning followed immediately by pithing to ensure permanent destruction of the brain. Intestinal segments were tied off before removal of luminal contents for enumeration of bacteria and phage.

### 2.7 Metagenomics sequencing and bioinformatics analysis

Raw fastQ files were quality-filtered (fastp v0.23.3) using a PHRED score of 30, and minimum length of 60 bp. Final read quality was assessed using fastQC v0.12.0 and multiQC v1.22.1. Pig reads (*Sus scrofa*, NCBI accession: GCA_000003025.6) were removed using HiSat2 v2.21 (Kim et al., 2019). The remaining reads were classified using Kraken2 (standard database) and Bracken v2.7.0 (Lu et al., 2017). Stacked bar charts of bacterial genera were produced in R (v.4.3) using ggplot2 (Wickham, 2016). Ecological diversity metrics were calculated using Vegan (Dixon, 2003). Beta-diversity was visualized using NMDS based on Bray–Curtis dissimilarity. Significant differences between metagenomes were determined using PERMANOVA (999 permutations).

### 2.8 Statistical analysis

Statistical calculations were performed using R v4.3, with figures prepared using ggplot2. Bacterial and phage counts were log_10_-transformed, and clinical scores were aggregated per animal before calculating group means and standard deviations. Differences in bacterial and phage counts between phage-treated and challenge groups (*n* = 5) were determined using the Kruskal–Wallis test followed by Dunn’s post-hoc test. The same tests were applied to weight and rectal temperature data. Clinical scores were compared using the Chi-Square test.

## 3 Results

### 3.1 Susceptibility of enteric bacteria to bacteriophage infection

The phages used in this study were tested for their ability to infect a range of target and non-target Gram-negative enteric bacterial species (Supplementary Table S2). The phage were genus-specific, exhibiting no detectable cross-infection of non-target genera. Phage Phi10 infected *Salmonella* serotypes Enteritidis and Typhimurium but not Infantis, likely reflecting shared O-antigens between the first two serotypes. Phage P433.1 could not infect the other serotypes of *E. coli* tested, representing diverse pathogenic and non-pathogenic isolates. The one-step growth curves showed latent periods of 15, 40, and 60 min for phages P433.1, Phi10, and LL-A6, with corresponding burst sizes of ~240, 20, and 100 respectively (Supplementary Figure S1).

### 3.2 Viability of bacteriophage in simulated gastric fluid

The *in vitro* assay examining phage viability in SGF revealed that adding CaCO_3_ (in a concentration equivalent to the *in vivo* trial) had a protective effect against the low pH of the simulated gastric fluid, showing similar recovery to the control for all three phages after 1 h incubation. However, in the absence of CaCO_3_, phage titres fell by >5 log_10_ PFU/mL below detectable limits (2 log_10_ PFU/mL) (Supplementary Figure S2) by 1 h.

### 3.3 Phage genomes reveal inhibitors of bacterial defense systems and absence of AMR and virulence genes

The genome sequences of phage P433.1, Phi10 and LL-A6 are available from NCBI GenBank under accessions MN384978, MN384979 and PQ141064 respectively, with genome maps presented in Supplementary Figures S3–S5. ETEC phage P433.1 is a *Vectrevirus*, closely related to K1-5 (NC_008152). It encodes an Ocr-like antirestrictase, a potent inhibitor of the BREX bacteriophage exclusion defense system (Isaev et al., 2020). *Salmonella* phage Phi10 is a *Kuttervirus*, closely related to vB_SenM-2 (KX171211.1), and also encodes an Ocr-like antirestrictase. *C. coli* phage LL-A6 is a *Firehammervirus*, closely related to CP21 (NC_019507). None of the phage harbored genes associated with lysogeny, virulence (Virulence Factor Database; Chen, 2004) or antimicrobial resistance (Comprehensive Antibiotic Resistance Database; Alcock et al., 2019).

### 3.4 Bacteriophage reduced *S.* Typhimurium and ETEC colonization of weaned piglets

Neither the bacterial pathogens used in the challenge, nor bacteriophage which infect them could be detected in any of the pigs prior to commencement of the trial.

**Figure 2:**
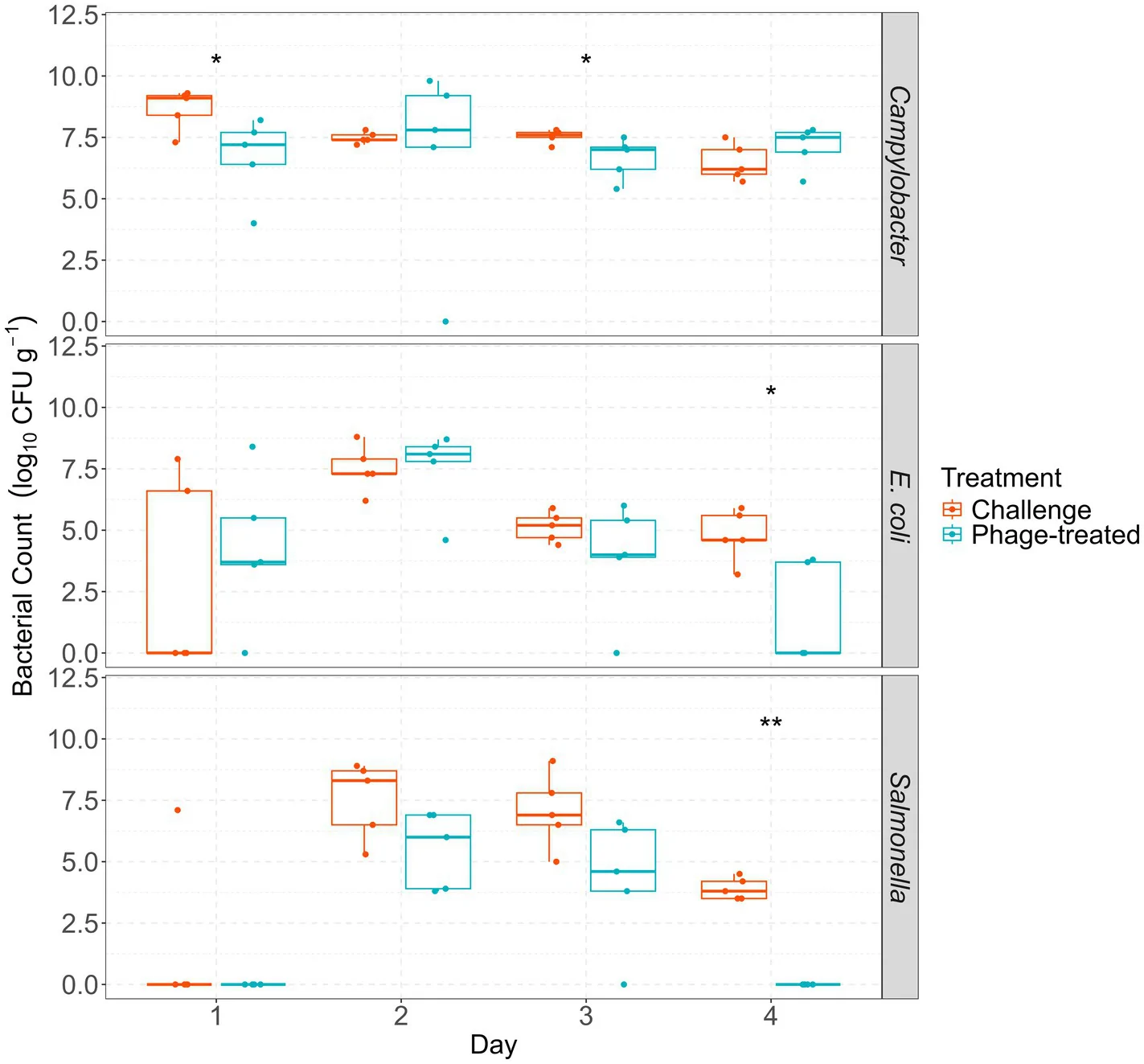
Faecal counts of ETEC, *S.* Typhimurium and *C. coli* in phage-treated piglets (log_10_ CFU/g). Each animal was infected orally with 10mL mixture of ETEC P433, *S.* Typhimurium 4/74 and *C. coli*, each at ~9 log_10_ CFU/mL. Four hours post-challenge the pigs in the phage-treated group were dosed orally with 10mL of a cocktail of phage P433.1, Phi10 and LL-A6, each at ~10 log_10_ PFU/mL. Phage treatment (or an equal volume of buffer for the control group) was repeated daily until the end of the trial. Bacteria CFU counts are plotted individually along with the median value per group (—). Significant differences between groups: *p<0.05, **p<0.01.

Weaned piglets in the phage-treated group were challenged simultaneously with ETEC, *S.* Typhimurium and *C. coli*, then treated with phage 4 h later and at 24 h intervals thereafter. Pigs in the challenge and sentinel groups were given the pathogen challenge only, or buffer only. Fecal counts of each pathogen in the phage-treated and challenge groups are presented in Figure 2. Pigs in the sentinel group remained negative for all three pathogens and their bacteriophage throughout.

Median CFU counts of both ETEC and *Salmonella* peaked 48 h after challenge in both phage-treated (7.3 and 8.3 log_10_ CFU/g) and challenge groups (8.1 and 6.0 log_10_ CFU/g). Although counts in both groups subsequently declined, median ETEC and *Salmonella* CFU in the phage-treated group were significantly lower (>1.8 log_10_ CFU/g, *p* < 0.05) than the challenge group by the end of the trial, with most CFU counts below the limit of detection (<2.0 log_10_ CFU/g). Conversely, phage treatment had little effect on *Campylobacter*, with median counts in the challenge group declining from 9.1 to 6.2 log_10_ CFU/g and numbers in the phage-treated group remaining between 7 and 7.8 log_10_ CFU/g.

**Figure 3:**
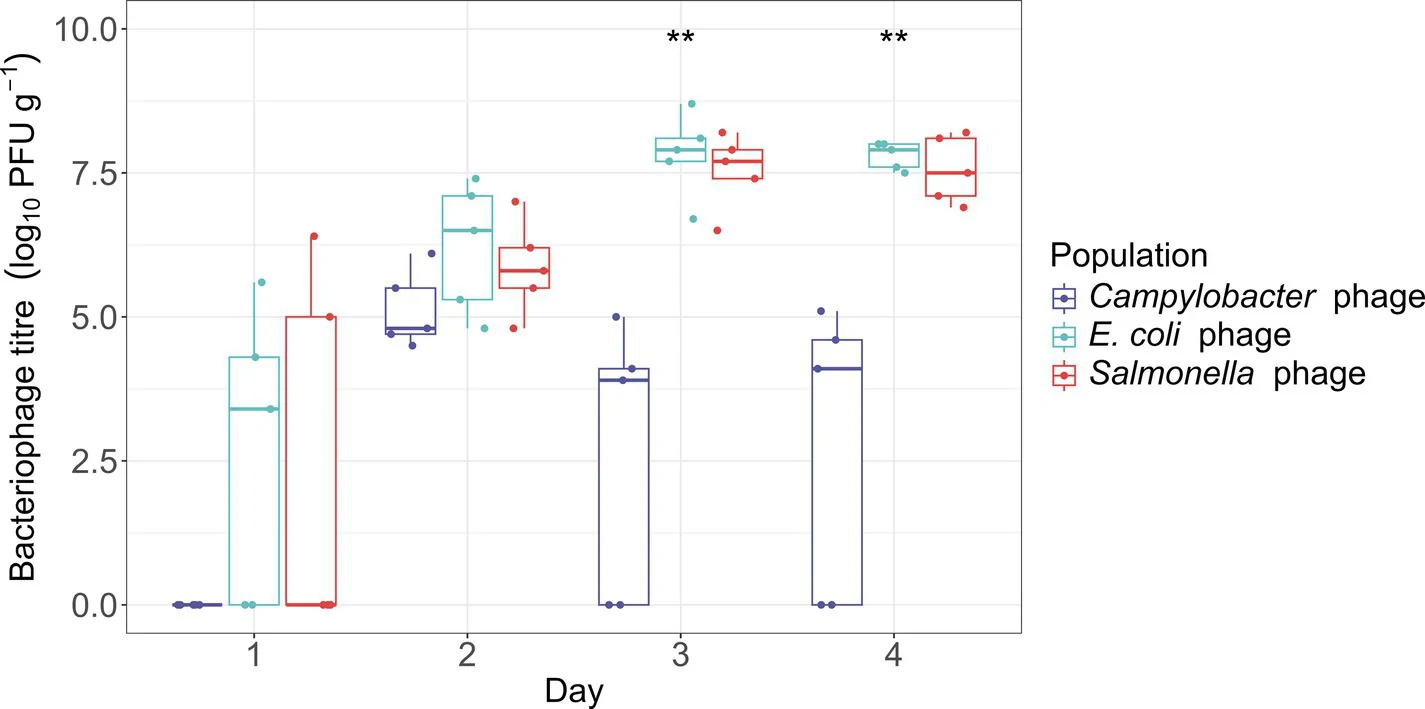
Faecal counts of phage P433.1, Phi10 and LL-A6 in phage-treated piglets (log_10_ PFU/g). Pigs were dosed orally with 10mL of a cocktail of phage targeting ETEC (P433.1), *S.* Typhimurium (Phi10) and *C. coli* (LL-A6), each at ~10 log_10_ PFU/mL. Phage treatment (or an equal volume of buffer for the Challenge-only group) was repeated daily until the end of the trial. Phage titres are plotted for individual animals along with the median value per group (—). Significant differences between groups: **p<0.01.

Bacteriophage counts from pigs in the phage-treated and challenge groups are presented in Figure 3. Titres of all phage increased 24 h after initial inoculation. However, titres of *Campylobacter* phage LL-A6 remained between 3.9 and 4.8 log_10_ PFU/g thereafter, whereas phages P433.1 and Phi10 were both significantly higher (*p* < 0.01) for days 4–5 (7.5–7.9 log_10_ PFU/g).

Median counts of bacterial pathogens and phage in different gut compartments following euthanasia on day 5 are presented in Table 1. Median counts were used as several groups contained bacterial or phage counts below the limit of detection, which did not conform to a normal distribution. Bacterial counts in the duodenum, jejunum and ileum were frequently below detectable limits for all three pathogens. Phage treatment was associated with significant reductions (>1.7 log_10_ CFU/g) in both ETEC and *S.* Typhimurium counts in the caecum and colon compared with the challenge group. Conversely, there was no significant difference in *Campylobacter* counts between the phage-treated and challenge groups. Coliphage P433.1 was detected in all intestinal segments, but present in greater numbers in the colon and caecum. Similarly, although *Salmonella* phage Phi10 was detected in the ileum, it was recovered in greater numbers from the colon and caecum. *Campylobacter* phage LL-A6 was recovered sporadically from the ileum, colon and caecum at up to 5.2 log_10_ PFU/g in the ileum. Animals in the sentinel group tested negative for bacterial challenge pathogens and therapeutic phage throughout the study.

**Table 1:** Bacteria and phage counts from pig intestinal segments following phage treatment.

Segment	*C. coli*		ETEC		*S. T*yphm		*Campylobacter* phage		Coliphage		*Salmonella* phage	
	Chal	Trt	Chal	Trt	Chal	Trt	Chal	Trt	Chal	Trt	Chal	Trt
Duodenum	0.0^†^	0.0^†^	0.0^†^	0	0	0	0	0	0	2.9	0	0
Jejunum	0.0^†^	0	0.0^†^	0.0^†^	0	0	0	0	0	3.1	0	0
Ileum	0.0^†^	0.0^†^	0.0^†^	0.0^†^	0	0	0	0.0^†^	0	3.7	0	3.6
Colon	6.8	5.5	3.7	0.0*	3.8	0.0**	0	0.0^†^	0	7.3	0	7.4
Caecum	5.7	5.5	4.1	0.0**	4.8	0.0**	0	0.0^†^	0	6.9	0	7.7

^†^Above the limit of detection for some animals in the group. *p < 0.05; **p < 0.01. Median counts (log_10_ CFU or PFU/g) of bacteria (ETEC P433, *S.* Typhimurium 4/74 and *Campylobacter coli* LL-SB7) and phage (ETEC phage P433.1, *Salmonella* phage Phi10 and *Campylobacter* phage LL-A6) respectively in segmented intestinal contents of pigs treated (Trt) or untreated (Chal) with bacteriophage following pathogen challenge. Counts are given for pigs at the end of the trial (day 5).

**Figure 4:**
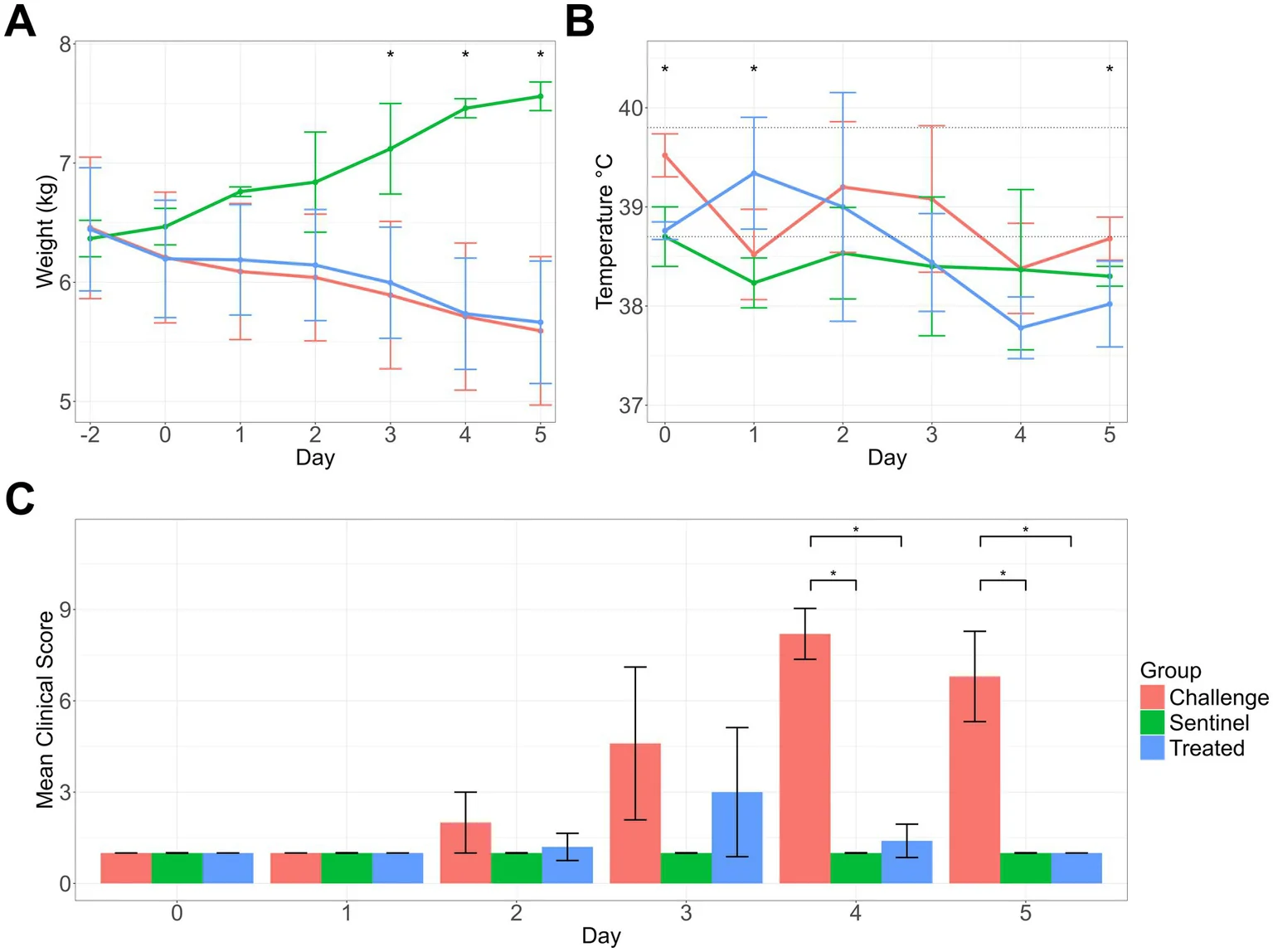
Physiological parameters and clinical scores of pigs challenged with three pathogens and treated with bacteriophage. Pigs in the challenged and phage-treated groups (*n*=5) were inoculated orally with a 10mL mixture of ETEC P433, *S.* Typhimurium 4/74 and *C. coli*, each at ~9 log_10_ CFU/mL. Four hours post-challenge the pigs in the phage-treated group were dosed orally with 10 mL of a cocktail of phage P433.1, Phi10 and LL-A6, each at ~10 log_10_ PFU/mL. Phage treatment (or an equal volume of buffer for the control group) was repeated daily until the end of the trial. Sentinel pigs (*n*=3) remained unchallenged and untreated throughout. Panels A, B and C show mean weights, rectal temperatures and cumulative clinical scores (±s.d.) of the three groups respectively. In Panel B, the horizontal lines represent the typical rectal temperature range for pigs (https://www.msdvetmanual.com/multimedia/table/normal-rectal-temperature-ranges). Clinical scores evaluated faecal consistency, general condition, appetite and depression. Significant differences between groups: *p<0.05.

The reductions in ETEC and *Salmonella* in phage-treated pigs corresponded with significant reductions in mean aggregated clinical scores on days 4 and 5 compared with the challenge group (Figure 4C). Although the clinical scores of the phage-treated group were numerically higher than the sentinel group, this difference was not significant on any day. The weights and rectal temperatures of both the challenged and treated pigs decreased over the trial (Figures 4A,B). Conversely, the sentinel group increased in weight significantly (*p* < 0.05) compared with the other groups from day 3 onwards. Rectal temperatures in the challenge and treated groups were generally higher but exhibited more daily fluctuation than the sentinel group in the first 3 days before converging by the end of the trial. All three groups were below the typical temperature ranges for pigs at various points.

### 3.5 Phage therapy had no significant detectable effect on the fecal microbiome

Metagenomic reads from this project are available from NCBI (BioProject: PRJNA1162153). Shotgun metagenomics was used to determine the effect of pathogen challenge and phage treatment on the pig gut microbiota. The metagenomic DNA from pig fecal samples was extracted from challenged and phage-treated pigs on days 2, 4, and 5, and sentinel pigs on day 5, and subjected to Illumina sequencing. There was no significant difference in alpha diversity metrics between pigs from different treatment groups, with the Shannon, Simpson and Inverse Simpson indices ranging between 2.80–3.10, 0.92–0.95, and 13.10–18.40, respectively (Supplementary Figure S6).

**Figure 5:**
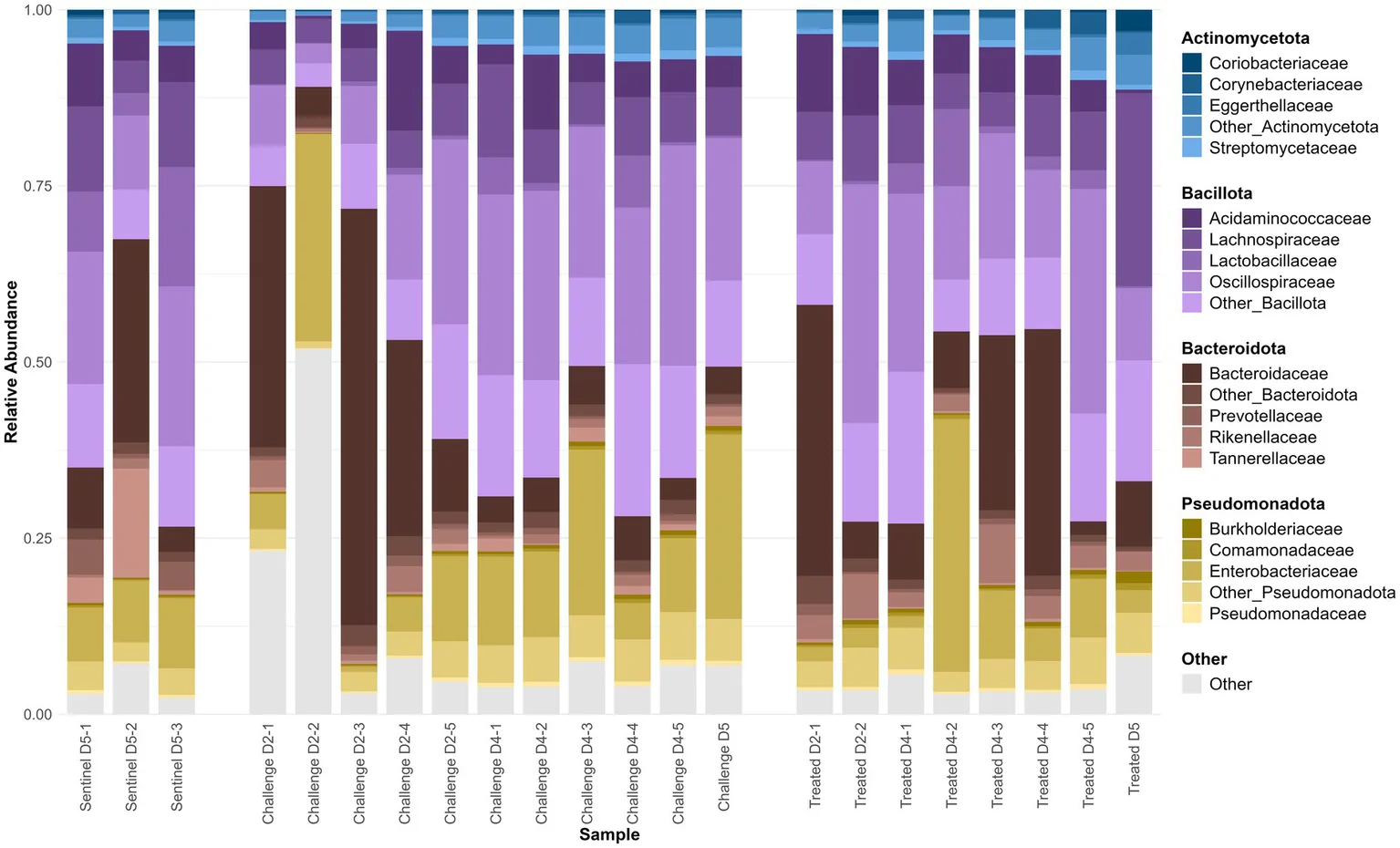
The plot represents the most abundant bacterial genera in pig faecal samples, grouped by phylum, as determined by metagenomic analysis using Kraken2 and Bracken. Faecal samples were taken from weaned pigs either challenged with ETEC P433, *S*. Typhimurium 4/74 and *C. coli* LL-SB7 (“Challenged”); challenged and treated with bacteriophage P433.1, Phi10 and LL-A6 (“Treated”); or remained unchallenged sentinel pigs (“Sentinel”). The samples were taken from individual pigs two, four and five days after bacterial challenge (D2, D4 and D5 respectively).

The bacterial communities are presented in Figure 5. The pigs in the challenge group harbored a greater abundance of *Enterobacteriaceae* reads than the phage-treated group (apart from Treated D4-2). *Bacteroidaceae* abundance varied substantially within both challenged and phage-treated groups over the course of trial, comprising >50% of reads in one pig and 25–33% of reads in six more. Conversely, *Pseudomonadaceae* abundance was largely consistent across both sentinel and treatment groups. Aggregating mean abundance values per group and day revealed more nuanced differences between the groups at the genus level (Supplementary Figure S7). Challenge-group pigs showed greater *Escherichia* abundance, whereas *Bacteroides* was enriched in phage-treated pigs.

**Figure 6:**
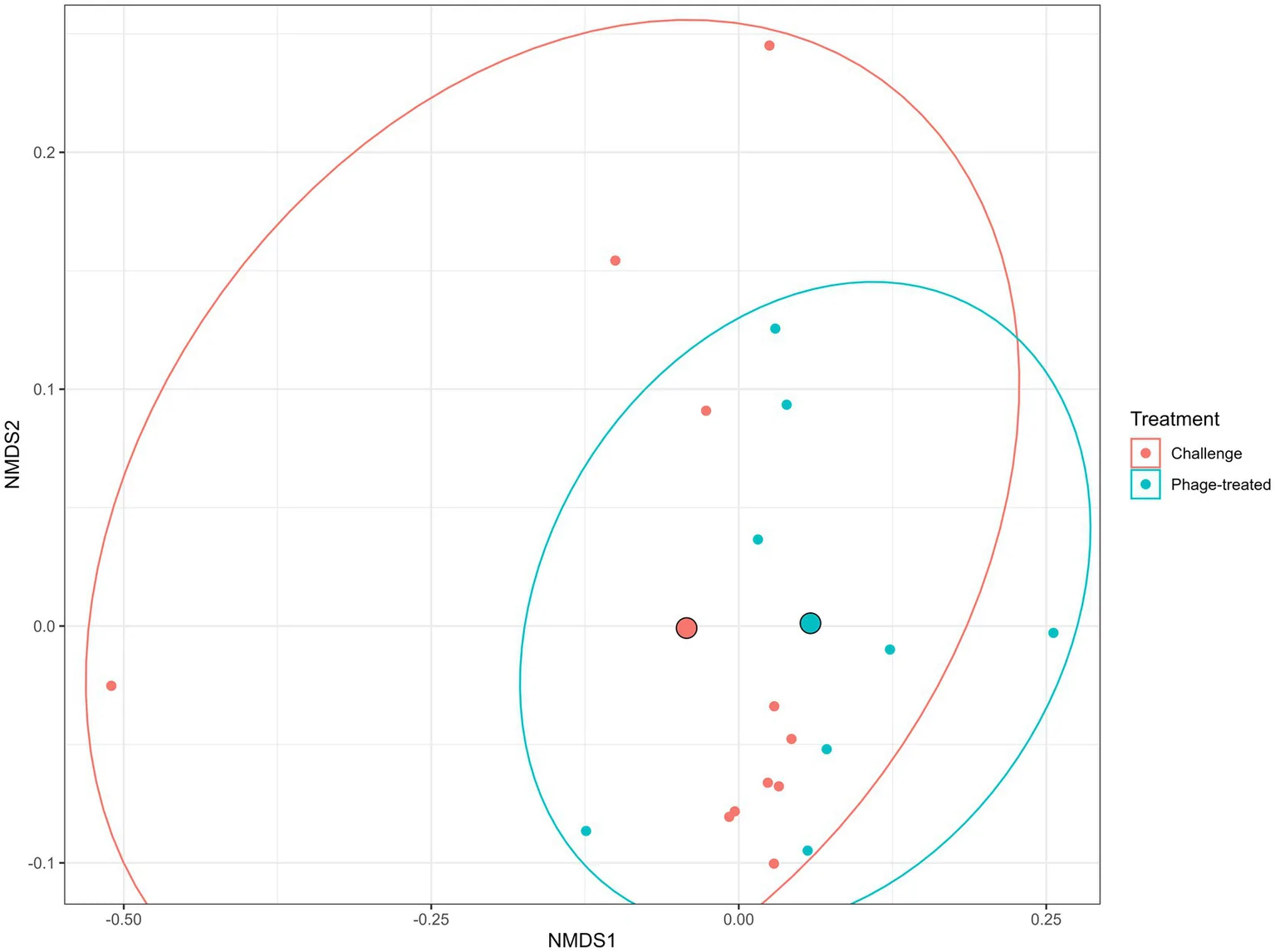
NMDS plot of individual pig microbiome beta diversity scores. Metagenomic DNA was extracted from the faeces of pigs which were either challenged with a mixture of three pathogens (ETEC P443, *S.* Typhimurium 4/74 and *C. coli* LL-SB7) alone, or challenged then treated with a bacteriophage cocktail targeting these pathogens. Metagenomic reads were subtractively-mapped against the pig genome before taxonomic classification using Kraken2. Genus-level assignments for each sample were extracted using Bracken, followed by the calculation of a Bray-Curtis distance matrix using Vegan in R. NMDS plots were prepared using ggplot. Ellipses and centroids (large dots) for each treatment group were manually calculated in R.

Beta diversity was calculated using the Bray-Curtis index and visualized using a Non-metric Multidimensional Scaling (NMDS) plot (Figure 6), which indicates considerable overlap between the pigs in the challenge and phage-treated groups. Permutational Multivariate Analysis of Variance (PERMANOVA) determined that the differences between the groups were not significant (*p* = 0.5).

## 4 Discussion

This study reports the first investigation of phage therapy using weaned pigs as a model of polybacterial enteric infection in young children. The weaned pig model used here successfully represents the mixed bacterial infections which often occur in LMIC where hygienic conditions are suboptimal (Hawash et al., 2017; Liu et al., 2016; Shah et al., 2016) and where contaminated water, for example, may introduce mixtures of enteric pathogens. A recent report has indicated that similar to the earlier results by Smith and Huggins (1983) and Smith et al. (1987) administration of phages, in that case via bedding, was able to reduce the intestinal counts of a single ETEC strain and improve clinical signs (Mahmud et al., 2026).

In the present study, administration of a cocktail of lytic bacteriophages targeting three major enteric bacterial pathogens was associated with significant improvements in the clinical condition of the pigs. Phage-treatment also resulted in a significant reduction in the numbers of ETEC and *S.* Typhimurium, and a concomitant increase in the titres of the specific phages infecting these bacteria. There have been few trials of bacteriophages against enteric pathogens in humans. There are historical reports of the use of phage against dysentery but many of these early reports are difficult to analyze in depth and come from an era where controlled experimentation was poor (Orlova and Garnova, 1970). The use of bacteriophage cocktails should be routine where more than one bacterial host is targeted. It may also be used to combat phage resistance during infection and treatment, with two phages being administered, the first to kill the bacterial strain and the second to kill any resistant mutants that arise in response to the first phage (Smith et al., 1987).

In contrast, *Campylobacter* colonization was largely unaffected by phage LL-A6. This is likely due to inadequate phage replication, as median titres of LL-A6 were ~3 log_10_ PFU/g lower than the other phage, with counts in several animals below the limit of detection. We also know from previous work (Barrow et al., 1998; Berchieri Jnr. et al., 1991) that when the numbers of the target bacterial strain fall below a certain threshold the likelihood of contact between phage and target bacterium is greatly reduced to the extent that that phage cannot establish itself and is lost with no concomitant reduction in bacterial numbers. The poor replication of LL-A6 may also reflect limited access to actively growing *C. coli*, which is adapted to penetrate deep into the mucus layer, and may preferentially colonize the caecum and colon; regions producing more mucus than the small intestine (Mdegela et al., 2011). Conversely, ETEC and *S.* Typhimurium both colonize the small intestine, where mucus coverage is thinner (Cevallos-Almeida et al., 2018; Dubreuil et al., 2016) in addition to the caecum and colon. ETEC colonizes the intestine by adhering to the mucosa (Jones and Rutter, 1972) and *S.* Typhimurium multiples optimally in close association with the mucosa where nutrients and oxygen levels, as electron acceptors, are highest (Harvey et al., 2011).

In the present study, the time period between pathogen challenge and phage treatment was 4 h. Although early mixing in the stomach with phage activity was conceivable, this is unlikely to account for the results. Feed was withheld prior to both bacterial challenge and phage treatment, resulting in faster gastric transit. Experimental estimates indicate 50% of liquid feed is cleared from the pig stomach within 1.4 h (Davis et al., 2001), suggesting that any mixing of phage and bacterial suspensions in the stomach after 4 h would be minimal. Furthermore, gastric lysis cannot explain why *Salmonella* and ETEC counts were reduced, but *Campylobacter* numbers remained relatively stable.

All three phages maintained their titres in a simulated gastric environment over 1 h in the presence of antacid, but fell below detectable levels when CaCO_3_ was omitted. The use of antacid alongside phage therapy may pose challenges in LMIC as this may undermine an important infection barrier although treatment of an acute infection is likely to have priority over infections which may not yet have taken place. RNA bacteriophages have been found to remain highly effective in the intestine with simultaneous antacid administration (Colom et al., 2019). However, bacteriophage can be protected using alternatives that do not compromise gastric acidity, for example encapsulation in alginate–carrageenan or chitosan-alginate microbeads (Dlamini et al., 2023; Malik et al., 2023). These methods could also better target phage release to the main site of infection in the gut.

The weights and rectal temperatures of pigs decreased in both phage-treated and challenged groups throughout the trial, in contrast to the sentinel group which continued to gain weight. Post-weaned pigs often lose weight for 4–5 days because of stress (Campbell et al., 2013). Additional animal handling and pathogen challenge may have exacerbated this trend. The scores for enteritis for the phage treated group were significantly better than those for the Challenge-only group. Rectal temperatures were more similar, and converged toward the end of the trial, though at a lower level. This may reflect the timing of temperature readings, as heat production is closely tied to circadian rhythm in pigs (McCracken and Caldwell, 1980), with up to a 0.9 °C difference reported across the day (Lord et al., 1999).

Although international institutions are advocating new approaches to tackling antimicrobial resistance including the use of phage (OECD, 2022; Pelfrene et al., 2016; Review on Antimicrobial Resistance, 2014) there remain hurdles relating to the treatment of infections from which several different bacterial pathogens may be isolated in addition to viral pathogens, the development of phage resistance, phage stability, the limitations associated with the relatively high specificity of most phages, effect on the microbiome and safety some of which have been reviewed recently (Venhorst et al., 2022; Wang et al., 2025).

Fecal microbiome analysis revealed appreciable variability between individual samples, even within the same treatment group, as reflected by the alpha diversity values (Mahmud et al., 2026). Environmental, host genetic and dietary factors are known to affect microbiome content (Kubasova et al., 2018; Pedersen et al., 2013). Disruption of the microbiome following challenge with three bacterial pathogens would be expected in both groups as reported previously (Rhouma et al., 2021). The NMDS beta diversity plot showed substantial overlap between phage-treated and challenge groups suggesting that bacteriophage treatment did not result in any further changes to the fecal microbiome following bacterial challenge. However the relatively small group sizes mean that only large differences between the groups are likely to be detected. Despite this, the finding that phage treatment did not produce a significantly different microbiome from either challenge only or sentinel pigs is encouraging, although the long-term consequences for pig and human health and productivity remain unknown. There are reports that the presence of high numbers of phage the alimentary tract can improve executive function but how this relates to the changes in the microbiome is unclear (Mayneris-Perxachs et al., 2022). Given that polyvalent phages which infect both *Salmonella* and *E. coli* have been isolated from pigs previously (Sui et al., 2021), the phages used in this study may be able to infect non-target bacterial species depending very much on the receptors. However, *in vitro*, these phages were unable to infect a panel of common enteric bacteria, nor cross-infect the pathogens used in the experimental challenge. This does not preclude infection of other strains, but suggests that effects on the microbiome are more likely to be localized and limited. Phage Phi10 infected strains of *S.* Enteritidis and *S.* Typhimurium, but not *S.* Infantis likely due to shared O-antigens 1 and 12 in the former serotypes, which may be phage receptors. Phage P433.1 was unable to infect the other *Enterobacteriaceae*, including 14 *E. coli* strains representing eight O-serotypes, suggesting limited impact on commensal non-target bacterial populations.

In phage therapy the relatively high degree of specificity remains a persistent issue. In the seminal papers by Smith and Huggins, those authors sought phages for which the K88 and K99 surface antigens were receptors such that a range of serotypes possessing these surface virulence determinants could be targeted, again with the rationale that phage-resistant mutants would mostly have lost their receptor and therefore be avirulent (Barrow et al., 1998; Smith and Huggins, 1983). They were unsuccessful in this particular search but used phages which attached to LPS instead. Phage-resistant rough mutants which emerged from this experiment were found to be slightly less virulent. Many *E. coli* and *Salmonella* strains are now multi-resistant, with incompatibility group F plasmids predominating in human medicine. The approach advocated by Colom et al. (2019) and Ojala et al. (2013), where the plasmid transfer apparatus is targeted by phages specific for the sex pili produced by these plasmids, might be a more effective approach to reducing the limitations of phage specificity since any serotype possessing an IncF plasmid might be targeted under the right circumstances (Atterbury et al., 2026).

In summary, this study demonstrates that phage therapy can effectively reduce colonization of two human and zoonotic bacterial pathogens in pigs, with associated improvements in clinical scores. Phage therapy was not associated with significant changes in the gut microbiome. These findings support our central hypothesis that phage therapy can selectively reduce the burden of more than a single bacterial pathogen at once without disrupting commensal populations in the pig gut. This suggests that phage therapy may be preferable to broad-spectrum antibiotics for the selective reduction of bacterial pathogens during MSD in children in LMIC, while minimizing disruption to commensal gut populations, and it clearly merits further investigation.

Several limitations of this study should also be considered. First, the relatively small group sizes and short duration of the study may limit the generalizability of the findings. Second, the absence of a group treated with bacteriophage alone precludes assessment of phage viability in the absence of target hosts. Third, although shotgun metagenomic sequencing enabled comparison of changes in major bacterial families and genera between treatment groups, it lacks the resolution needed to detect changes in minor bacterial populations. Future studies addressing these limitations will be important to validate and extend these findings.

## Data Availability

The datasets presented in this study can be found in online repositories. The names of the repository/repositories and accession number(s) can be found in the article/Supplementary material.

## Footnotes

1
https://www.un.org/pga/71/wp-content/uploads/sites/40/2016/09/DGACM_GAEAD_ESCAB-AMR-Draft-Political-Declaration-1616108E.pdf

4
arriveguidelines.org
