# Supplementary material for: The pig as a preclinical model for human phage therapy of polybacterial enteric infections
Source: Front Microbiol. 2026 Aug 7;17:1857948. doi: 10.3389/fmicb.2026.1857948 (PMC13493500; doi:10.3389/fmicb.2026.1857948)
Supplement: Supplementary file 1 [file Table_1.DOCX]

Supplementary Material

**B**

**Supplementary Figure S1. Bacteriophage one step growth curves.** Liquid cultures of *E. coli* P433 (A), *Salmonella* Typhimurium 4/74 (B) and *C. coli* LL-SB7 (C) were separately infected with phage P433.1, Phi10 or LL-A6. Free phage titres were recorded in samples taken from infected cultures at intervals, with mean and standard deviation values plotted from three biological and three technical repeats.

**A**

**Supplementary Figure S2. Viabilty of bacteriophage in a simulated gastric environment.** Bacteriophage (10^9^ PFU/mL) were diluted 1:9 in simulated gastric fluid (3.2 mg/mL pepsin, 0.5% NaCl, pH2) or SM buffer (pH7) to which was added 0.1mL of 20% w/v CaCO_3_ (treated) or SM buffer (control) and incubated for 1 h at 37°C before enumerting phage on double agar overlays seeded with their respective host strains. Data represents mean recovery (± SEM) based on three biological and three technical repeats.

**Supplementary Figure S3. *Salmonella* Phage Phi10 genome 159,043 bp schematic genomic organization.** The purple arrows on the outer ring shows putative open reading frames, these with known function are labelled with arrowheads. The inner rings represent GC content and GC skew in the genome.

**Supplementary Figure S4. *E. coli* Phage P433.1 genome 43,865 bp schematic genomic organization.** The purple arrows on the outer ring shows putative open reading frames, these with known function are labelled with arrowheads. The inner rings represent GC content and GC skew in the genome.

**Supplementary Figure S5. *C. coli* Phage LL-A6 genome 166,199 bp schematic genomic organization.** The purple arrows on the outer ring shows putative open reading frames, these with known function are labelled with arrowheads. The inner rings represent GC content and GC skew in the genome.

**Supplementary Figure S6: Scatter plot of pig microbiome alpha diversity scores.**

Metagenomic reads from the faeces of pigs challenged with *E. coli* P443 and *S.* Typhimurium 4/74 and treated (Phage-treated) or untreated (Challenge) with bacteriophage were subtractively-mapped against the pig genome before taxonomic classification using Kraken2. Genus-level assignments for each sample were extracted using Bracken, and alpha diversity calculations performed using Vegan in R. Metagenomic reads from the faeces of pigs unchallenged and untreated (Control) are provided as a comparison.

**Supplementary Figure S7: Stacked bar plot of relative abundance of bacterial genera in pig faeces.**

The plot represents the relative abundance of bacterial genera (determined using metagenomic sequence reads, followed by analysis using Kraken2 and Bracken) in faecal samples of weaned pigs treated either challenged with *E. coli* P433 and *S.* Typhimurium 4/74 (T1), Challenged and treated with bacteriophage P433.1 and Phi 10 (T2) or unchallenged and untreated (C). The samples were taken from individual pigs two, four and five days after bacterial challenge (D2, D4 and D5 respectively).

**Supplementary Table S1: Clinical symptom scoring system used for pigs**

**General condition**

0 = Clinically healthy appearing animal

1 = Slightly abnormal condition

2 = Moderately abnormal condition

3 = Severely abnormal condition

**Appetite**

0 = Animal is eating normally

1 = Reduced appetite, picking at feed, feeding less vigorously

2 = Animal is completely off feed

**Depression**

0 = Normal. Pig is up and active when entering the room or when approaching.

1 = Mild depression. Pig is slightly inactive but does get up after being approached. Pig moves slowly and appears lethargic; shows some interest in food and social interaction, but only stays active for a few minutes; animal may appear gaunt and have rough coat.

2 = Moderate depression. Pig shows pronounced inactivity and only gets up after light back pressure. Pig displays little or no interest in food and/or social interaction; pig tends to lay back down almost immediately and/or stands with head down; pig is noticeably gaunt, and coat is rough. Pig staggers and appears slightly uncoordinated at the walk.

3 = Severe depression. Pig is moribund, unresponsive to stimuli, unable to get up or does so with difficulty; shows no interest in surroundings, food, or social interaction; appears anxious.

**Respiratory distress**

0 = None. Respiratory movements are subtle, superficial, and can only be observed when animal is quietly laying down.

1 = Mild. Respiratory movements are obvious and predominantly abdominal.

2 = Moderate. Respiratory movements are exaggerated, entirely abdominal, and laboured.

3 = Severe. Respiration is extremely laboured and entirely abdominal; Pig breathes through the mouth (continuously or intermittently); nose and/or ears are cyanotic.

**Coughing**

0 = No coughing during observation period

1 = Coughing infrequent (but more than once) or frequent during observation period

**Faecal consistency:**

The severity of diarrhoea will be based on the assessment of the fluidity of the faeces, as described below for faecal score (1 to 4):

1 - Normal, firm but not hard;

2 - Soft, does not hold form;

3 - Runny, spread - like pancake mix;

4 - Watery, liquid - like orange juice.

| Strain | *Campylobacter* phage LL-A6 | *E. coli* phage P433 | *Salmonella* phage Phi 10 |
| --- | --- | --- | --- |
| *Aeromonas hydophila* 36 (WT) | - | - | - |
| *Campylobacter coli* LL-SB7 | + | - | - |
| *Citrobacter freundii* NCTC 9750 | - | - | - |
| *Enterobacter cloacae* NCTC 13406 | - | - | - |
| *Escherichia coli* NCTC 86 | - | - | - |
| *Escherichia coli* P433 O20:K101, 987P | - | + | - |
| *Escherichia coli* H309 O6 | - | - | - |
| *Escherichia coli* H321 O6 | - | - | - |
| *Escherichia coli* H333 O6 | - | - | - |
| *Escherichia coli* B41 O101:K30,99 | - | - | - |
| *Escherichia coli* B44 O9:K30,99 | - | - | - |
| *Escherichia coli* B79 O101:K32,99 | - | - | - |
| *Escherichia coli* B117 O8:K85,99 | - | - | - |
| *Escherichia coli* S13 O8:K85,99 | - | - | - |
| *Escherichia coli* S14 O8:K85.99 | - | - | - |
| *Escherichia coli* P18 O8:K87,88 | - | - | - |
| *Escherichia coli* P15 O141:K85,88 | - | - | - |
| *Escherichia coli* P54 O147:K85,88 | - | - | - |
| *Escherichia coli* P56 O139:K82 | - | - | - |
| *Klebsiella aerogenes* NCTC 10006 | - | - | - |
| *Klebsiella oxytoca* NCIMB 11466 | - | - | - |
| *Proteus mirabilis* WT | - | - | - |
| *Pseudomonas aeruginosa* PA01 (WT) | - | - | - |
| *Salmonella* Infantis NCTC 6703 | - | - | - |
| *Salmonella* Enteritidis NCTC 13349 | - | - | + |
| *Salmonella* Typhimurium LT2 | - | - | + |
| *Salmonella* Typhimurium 4/74 | - | - | + |
| *Serratia marcescens* WT | - | - | - |
| *Yersinia enterocolitica* NCTC 10460 | - | - | - |

**Supplementary Table S2: Host range profiles of therapeutic bacteriophage.**

The sensitivity of 29 Gram negative enteric bacteria to infection with the phage used in the present study. Dilutions of each phage suspension were spotted in triplicate on to double agar overlay lawns seeded with approx. 10^7^ CFU/mL of each bacterium and incubated for 24 h before examining for plaques. Bacterial strains sensitive and insensitive to phage infection are indicated by + or – respectively.
